# Digital Informed Consent: Modernising Information Sharing in Surgery to Empower Patients

**DOI:** 10.1007/s00268-022-06846-w

**Published:** 2022-12-03

**Authors:** Simon L. Parsons, Prita Daliya, Phil Evans, Dileep N. Lobo

**Affiliations:** 1 Trent Oesophago-Gastric Unit, Nottingham University Hospitals NHS Trust, City Hospital Campus, Hucknall Road, Nottingham, UK; 2 Gastrointestinal Surgery, Nottingham Digestive Diseases Centre and National Institute for Health Research (NIHR) Nottingham Biomedical Research Centre, Queen’s Medical Centre, Nottingham University Hospitals NHS Trust and University of Nottingham, Nottingham, UK; 3 EIDO Healthcare Limited, Bridgford Business Centre, 29 Bridgford Road, Nottingham, UK; 4 MRC Versus Arthritis Centre for Musculoskeletal Ageing Research, Queen’s Medical Centre, School of Life Sciences, University of Nottingham, Nottingham, UK

## Abstract

**Background:**

Despite the 2015 Montgomery Ruling highlighting key requisites for informed consent, little has changed to modernise data-sharing and documentation of the consent process. It can be difficult to gauge patient understanding and address all patient concerns in time-limited appointments. We aimed to assess the feasibility of a digital information-sharing platform to support a move towards a digital informed consent process.

**Methods:**

All adult patients referred to a single centre with symptomatic gallstones were invited to use a digital information-sharing platform to support the informed consent process prior to their first surgical clinic appointment. The platform provided patients with multimedia information on gallstones and available treatment options. It recorded the time spent accessing information, asked patients multiple choice questions (MCQs) to allow a self-test of understanding, documented a summary medical history, and allowed free text for patient questions. This information was summarised into a clinical report to support outpatient clinic consultations.

**Results:**

Of the 349 patients registered to use the digital platform, 203 (58.2%) [165 (81.3%) female, mean age 47.6 years (range 19–84 years)] completed all modules necessary to generate a clinical report. Some 130 patients (64.0%) answered all 10 MCQs correctly and spent a mean of 18.7 min (range 3–88 min) reading the consent information. Most patient-reported medical histories were deemed to be accurate.

**Conclusion:**

Despite difficulties with access, resulting in drop-outs, patients welcomed the opportunity to receive information digitally, prior to their consultation. Patients described feeling empowered and better informed to be involved in decision-making.

**Supplementary Information:**

The online version contains supplementary material available at 10.1007/s00268-022-06846-w.

## Introduction

The UK National Health Service (NHS) long-term plan states that “The NHS will offer a ‘digital first’ option for most, allowing for longer and richer face-to-face consultations with clinicians where patients want or need it” [1]. Delivering routine care and information to patients digitally in their own homes have the benefit of saving resources and costs for the NHS. It is also more convenient for the patient, saving time and expense by avoiding unnecessary travel to hospitals.

The digital first option has been accelerated by the COVID-19 pandemic [2]. However, any move to provide healthcare at home should not simply abandon the patient to “no care” or “inferior care” where the necessary assessment of the patient cannot be performed properly. Digital healthcare needs to be safe, of high quality and maintain trust between patients and clinicians. A comprehensive and responsive online care of the patient should be the goal. It should also free-up the clinician’s time to have a meaningful face-to-face or virtual consultation.

Informed consent has traditionally been performed face-to-face with verbal sharing of information supplemented by written or other forms of information. The clinical consultation has many components, with informed consent being only a small part and often lasting only a few minutes. The use of technical jargon, the pressure of time and an assumption of understanding can hamper true shared decision-making between healthcare professionals and patients. Clinicians often spend their time giving information to patients, rather than listening to what matters to them and answering their questions [3].

The use of digital information for patients to read at their convenience, can enhance the informed consent process by sharing standardised and evidence-based information in a way that patients can understand easily, using diagrams and animations and other forms of accessible information such as translations, Easy Read, British Sign Language, or simply listening to somebody reading out the information. Sharing information in a format the patient prefers is recommended in the UK General Medical Council (GMC) guidance on decision making and consent [3].

If a patient can engage in this digital sharing of information prior to clinic attendance, there is potential to make the consultation more efficient. Clinicians can then spend their time listening to what matters to the patient [3, 4], answering their questions, and sharing that richer consultation, resulting in true shared decision-making.

The aim of this feasibility study was to pilot the use of a digital information-sharing platform to support the informed consent process, including detailing patient engagement with the informed consent process, documentation of self-testing in knowledge acquisition, and to determine the reliability of self-reported *versus* medically-reported patient health.

## Methods

### Study design and setting

This prospective feasibility cohort study was performed in a large tertiary NHS trust prior to the COVID-19 pandemic, between August 2016 and March 2018. All adult patients aged 18 to 85 years with symptomatic gallstones referred for consideration for elective laparoscopic cholecystectomy were invited to participate either in person or by postal invitation. Inclusion and exclusion criteria are described in Table 1.

**Table 1:** Inclusion, exclusion and withdrawal criteria

*Inclusion criteria*
Male or female adult patients aged 18 to 85 years at recruitment
Referred for consideration for an elective laparoscopic cholecystectomy for symptomatic gallstones
Originally listed for a laparoscopic cholecystectomy but subsequently converted to open cholecystectomy or a more complex biliary procedure (including but not exclusive to bile duct exploration, biliary drain insertion, biliary bypass)
*Exclusion criteria*
Individuals unable to, or those choosing not to engage with the multimedia process (including those lacking mental capacity, those without access to a multimedia device, and those unable to use a multimedia device unassisted)
Individuals unable to read or communicate in English without the presence of a translator
Patients undergoing another major non-biliary operation during the same operation as their cholecystectomy
*Withdrawal criteria*
Laparoscopic cholecystectomy not performed (surgery postponed, not deemed appropriate, no longer required, patient choice)
Change in clinical circumstances so that inclusion criteria no longer met
Participant request

Patients willing to participate were provided with a uniform resource locator (URL) by post, and later by either email or short message service (SMS), to an encrypted website where they had digital access to participant information and were able to provide virtual consent online to participate in the study. Participants registered on the site were given access to digital information about gallstones and the treatment options available, including laparoscopic cholecystectomy.

Information were available in several formats with all written content developed with input from the Plain English Campaign (PEC) to minimise the use of medical jargon and ensure readability. Voiceover options and font magnifier functionality were also available. All videos and animations were supported with subtitles, voiceover, and British Sign Language video interface.

In addition to preoperative information on gallstones and the treatment options, patients were invited to complete several online surveys. These included a self-reported preoperative health questionnaire, two electronic surveys on patient reported outcome measures (ePROMs), and a multiple-choice survey to allow knowledge self-assessment on gallstone disease and the treatment options available. The time patients spent accessing the information on gallstone disease and available treatments was also recorded. All preoperative questionnaires, patient self-test scores and the time spent accessing information were then summarised into a clinical document (Supplementary Fig. 1: Patient Health and Understanding Report—PHUR) which was available for both patients and clinicians during their very first outpatient clinical review. Data on ePROMs were analysed separately as part of a secondary study [5].

### Variables

Data on patient and hospital characteristics were collected, including patient age, sex, Charlson Comorbidity Index (CCI) [6], body mass index (BMI), and total length of hospital stay.

### Outcomes

1. Participant recruitment and experience of using the digital information platform as part of an informed consent process.
2. Time spent engaging with the available digital patient information in support of the informed consent process.
3. Proportion of correctly answered self-test questions in the self-test survey to assess understanding of the patient information.
4. Comparison of self-reported *versus* medically-reported patient health information to assess the reliability of patient-reported health reporting.

### Statistical analysis

Data were analysed using GraphPad Prism® version 8.3.0 (GraphPad Software LLC, San Diego, CA, USA.). Differences between groups were evaluated using either Fisher’s exact test or the chi-squared test for categorical variables and the Mann–Whitney U-test for continuous variables. Differences were considered significant at p < 0.05.

### Ethics, consent, patient public involvement and reporting

The study was performed as part of a larger PhD project sponsored by Nottingham University Hospitals NHS Trust, through a collaboration with EIDO Healthcare Limited and the Royal College of Surgeons of England. The study proposal was appraised by the confidentiality advisory group: 16/CAG/0045, with public and patient involvement, and ethics committee approval: 16/SW/0088. Informed consent to participate in the study was obtained from participants virtually on the digital platform. The study was registered with ClinicalTrials.gov (NCT02810860) and was conducted and reported in accordance with the Strengthening the Reporting of Observational Studies in Epidemiology (STROBE) guidelines [7].

## Results

### Participant recruitment and demographics

**Fig. 1:**
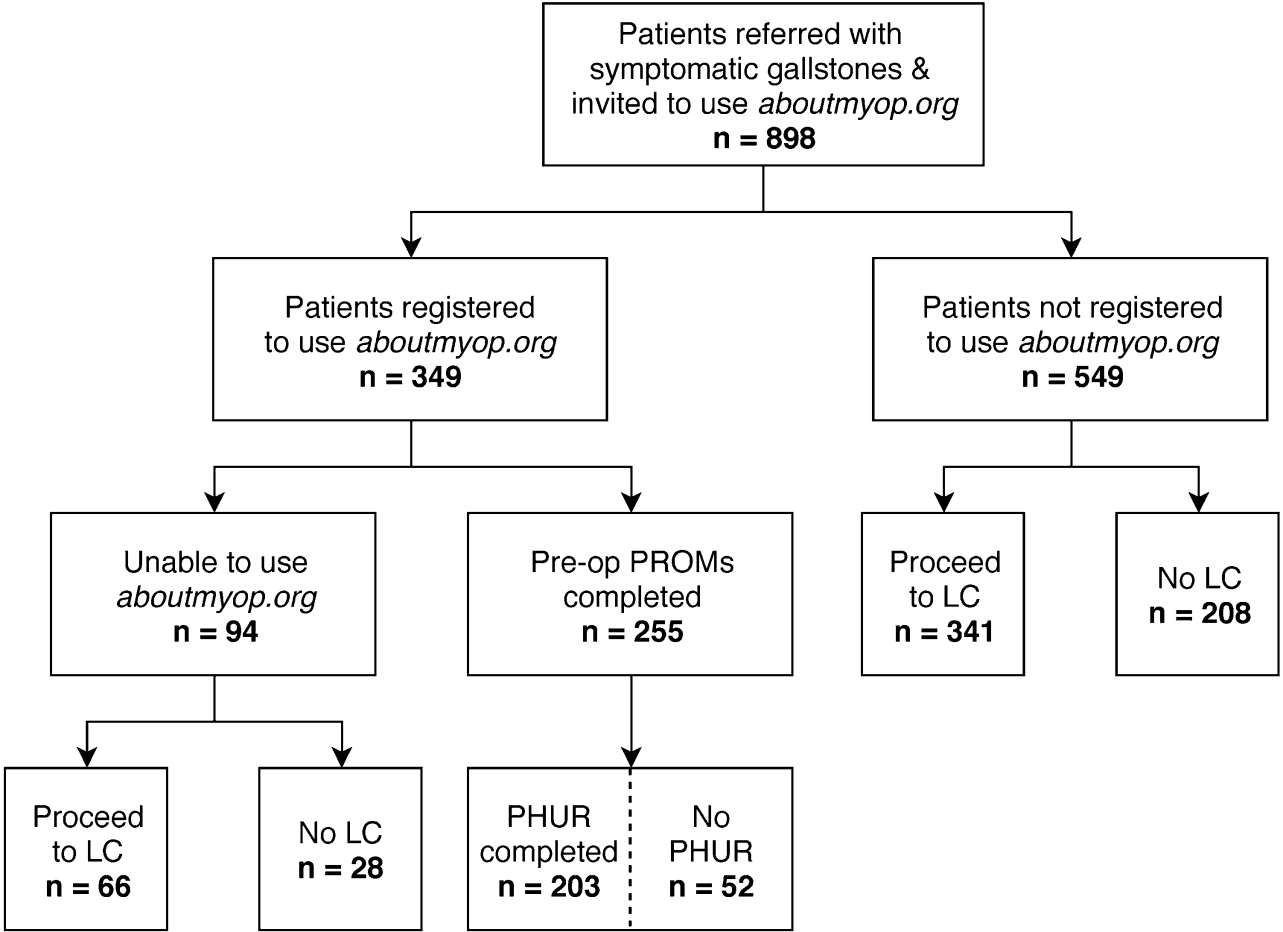
STROBE flow diagram. LC = laparoscopic cholecystectomy, PHUR = patient health and understanding report, PROMs = patient-reported outcome measures

A total of 898 eligible patients were invited to participate and use our digital interface. Of these only 349 (39%) patients completed the online registration to use the site. The preoperative surveys (preoperative health questionnaire & ePROMs surveys) were completed by 255 (28%), with 203 (23%) completing all pre-operative modules to generate the health report (Supplementary Fig. 1) available for both patients and clinicians (Fig. 1). Recruitment trajectory is shown in Supplementary Fig. 2 which also details a change in the rate of recruitment through the study period.

### Patient demographics

Study participants were younger than non-participants. There was no difference in sex, CCI, or BMI between study groups (Table 2).

**Table 2:** Participant demographics

	Participants *n* = 349	Non-participants *n* = 549	*p*-value
*Age (years)*			
Mean (SD)	47.6 (14.9)	51.8 (16.7)	< 0.001^*^
Range	19–84	19–85	
*Sex n (%)*			
Female	276 (79.1)	446 (81.2)	0.428^†^
Male	73 (20.9)	103 (18.8)	
*CCI n (%)*			
0	265 (75.9)	379 (69.0)	0.109^†^
1	43 (12.3)	79 (14.4)	
2	22 (6.3)	42 (77.8)	
≥ 3	19 (5.4)	49 (8.9)	
*BMI kg/m*^*2*^			
Median	29.7	29.8	0.951^‡^
IQR	25.5–35.5	26.2–34.7	

SD, standard deviation; IQR, inter quartile range; CCI, Charlson Comorbidity Index

^*^Mann–Whitney U-test, ^†^Chi-square test, ^‡^Wilcoxon signed-ranks test

### Patient health questionnaire

Self-reported information was derived from medical history questions completed by patients on the digital platform, whereas the medically-reported health information was derived from a conventional face-to-face consultation with a healthcare professional. There were no statistical differences in self-reported *vs*. medically-reported health information except for “liver disease”. Here, 33 out of 255 patients reported having liver disease which overestimated the true value of 16/255 according to the medically reported health information (Table 3).

**Table 3:** Self-reported versus medically-reported preoperative health

	Medical records	Self-reported	Self-report incorrect	*p* value (Fisher’s exact test)
*Myocardial infarction*				
Yes	5	5	1	
No	250	240	1	
Don’t know	0	10		> 0.99
*Congestive heart failure*				
Yes	2	1	1	
No	253	244	2	
Don’t know	0	10		> 0.99
*Peripheral vascular disease*				
Yes	2	3	3	
No	253	242	2	
Don’t know	0	10		0.68
*Chronic obstructive pulmonary disease*				
Yes	8	3	0	
No	247	249	5	
Don’t know	0	3		0.22
Diabetes mellitus				
Yes	17	14	0	
No	238	219	3	0.8
Don’t know		22		5
*Chronic kidney disease*				
Yes	7	15	13	
No	248	235	5	
Don’t know		5		0.08
*Tumour*				
Yes	13	16	6	
No	242	234	3	
Don’t know		5		0.57
*Liver disease*				
Yes	16	33	25	
No	239	217	8	
Don’t know		5		0.01

### Understanding of the consent information

The self-test survey to assess patient understanding of gallstone disease and treatment options was completed by 203 patients. Some 196 patients (96.6%) correctly scored 8 or more out of 10 (Table 4). The median time spent on the e-learning information was 17 min (range 3–88 min), as recorded by the digital platform. Answers to the health questions and the self-test survey for each patient were captured in the clinical report or PHUR along with the time the patient spent on the e-learning and any questions the patient wanted to ask the clinician (Supplementary Fig. 1).

**Table 4:** Patient responses to questions on consent

Question number	Question text	Number of correct responses [n (%)]	Number of incorrect responses [n (%)]
1	Where are gallstones formed?	202 (99.5%)	1 (0.5%)
2	What fluid are gallstones formed in?	201 (99.0%)	2 (1.0%)
3	What happens when gallstones move?	191 (94.1%)	12 (5.9%)
4	How are gallstones cured?	198 (97.5%)	5 (2.5%)
5	Which anaesthetic is used for the operation?	190 (93.6%)	13 (6.4%)
6	How can I help minimise complications following surgery?	190 (93.6%)	13 (6.4%)
7	What complications can occur after a cholecystectomy?	197 (97.0%)	6 (3.0%)
8	How can you recognise a serious complication?	179 (88.2%)	24 (11.8%)
9	When can you return to normal activities?	174 (85.7%)	29 (14.3%)
10	Who gives consent for the operation?	196 (96.6%)	7 (3.4%)

### Patient feedback

Feedback from patients regarding the digital platform as an adjunct to the consent process is summarised in Table 5.

**Table 5:** Participant feedback about the digital platform

**Pros**
Felt safe & secure to use
Registration went smoothly
Easy on the eye
Nice font
Easy to read from
Easy to navigate
Convenient to use in own time
Option to have a break & come back very useful
Flows well & is easy to follow
Concept & flow is excellent
Leap to multimedia very exciting
Language used is simple & easy to understand
Good use of diagrams & spot questions to keep user engaged
Great use of videos & visuals
Patient experience videos really reassuring
Process makes me feel responsible for making a good decision about my own healthcare
The PDF it spits out at the end is very clever
Informative
Reassuring to have some follow-up
**Cons**
Complicated log-in
Difficult to navigate log-in menu
Repeatedly locked out of log-in
Too many security checks to log-in
Some areas with too much text & information overload
Lacking white space in some areas
Could have used drop down menus
A lot of repetition
Difficult to use on mobile devices due to scrolling

## Discussion

This feasibility study explored some novel facets of the consent-process, with participating patients accepting a digital interface as an adjunct to their in person clinical consultation. Patients found the information easy to read and convenient, and liked the multimedia approach (Table 5).

### Participant recruitment

Although initial recruitment was poor, this was primarily due to the initial study set-up which required patient participants to type the study site URL into a web-browser to obtain access to the patient-facing digital platform. This was primarily due to stringent local information governance parameters, which were amended following further patient and public involvement, and further ethics committee approvals. Subsequent recruitment processes allowed patients to receive a hot link by either email or SMS with direct links to the study site and resultant improvements in participant recruitment (Supplementary Fig. 2). This emphasises the importance for digital platforms to be intuitive and easy to use.

A systematic review of digital tools for informed consent assessed understanding, satisfaction, anxiety and participation [8]. Digital technologies did not affect any outcome negatively and overall they had a positive effect, particularly in informed consent for clinical procedures when compared with informed consent for participation in research [8]. The authors also found that for clinical procedures, there was no benefit in the clinician being present during the digital sharing of information [8].

### Understanding of the consent information

The median time accessing the consent information was 17 min. This was similar to that found in a digital consent system utilised in orthopaedic surgery [9] and demonstrates good patient engagement. Patients demonstrated their understanding of the information required for informed consent by self-assessment, through answering 10 multiple-choice questions on gallstone disease and the available treatment (Table 4). Paragraph 27 of the new GMC guidance on consent states that patients need relevant information to be shared in a way they can understand and retain, so they can use it to make a decision [3]. Providing information in multimedia formats with interaction and teach-back techniques, as used in our digital platform, is the most effective way to help patients to retain information [10].

Digital information delivered on our platform allowed the patient to choose the place and time to engage with the information prior to attending clinic with no time pressure and no risk of coercion from the clinician, in accordance with the GMC guidance [3]. Coercion or pressure from the clinician was one of the findings of the Patterson inquiry in the UK, which recommended a two stage process of informed consent [11]. Furthermore, our digital platform gives complete traceability to the hospital in terms of what information was shared, the time the patient spent engaging, and questions answered, giving a measure of understanding. This ability to capture metrics associated with the consent procedure is a recognised benefit of digital consent [12] and could be important medicolegally [13, 14].

It is best to consider informed consent as two separate stages—the sharing of information and the clinical interaction where clinician and patient have a meaningful conversation leading to shared decision-making. Our study suggests sharing of information is best performed digitally and does not require the presence of a clinician, saving clinician time and, potentially, an unnecessary hospital visit for the patient. The second-stage does require both patient and clinician, and works best if the patient is pre-informed. The clinician confirms the procedure is recommended, confirms patient capacity to provide informed consent, and focuses on what matters to the informed patient, by answering relevant questions. This is also an opportunity for the clinician to adjust risks from the standard for that patient. This two-stage process with a pause to reflect [11] will become standard practice in the NHS [2]. The process is completed with a signed consent form (digital or paper), assuming the patient voluntarily decides to proceed. Digital consent solutions which take the patient and clinician through this process have been developed and are entering clinical practice [15].

These digitally supported systems allow the consultation to focus on “what matters to the patient” in the form of questions they wanted to ask the clinician. Since the Montgomery Ruling [4] the aspect of discussing “what matters to the patient” is a medicolegal imperative and is one of the 7 principles of informed consent defined by the GMC [3].

### Patient health questionnaire

Preoperative assessment is an important next step in the elective surgery pathway ensuring the patient is fit enough for the proposed surgery and that optimisation of their health can take place prior to surgery [16]. Digital preoperative questionnaires have been developed to screen patients so that only those who need to attend the preoperative assessment unit do so, with a view to saving time and resources [17]. In our study we compared patient self-reporting of health status with that performed by a healthcare professional, finding no statistical differences in most health-related questions. This raises the possibility of an automated patient-driven process as an adjunct or an alternative to routine processes. Further adequately powered studies are, however, required to determine if digital preoperative questionnaires can safely replace face-to-face preoperative assessment in a proportion of patients.

### Strengths and weaknesses

This feasibility study has provided strong evidence to support the process of informed consent, from measuring patient engagement, to providing a method for patients to self-assess knowledge acquired. It also demonstrates that patients can give a reasonably accurate self-report of their medical conditions. However, the relatively low number of registrations compared with those invited (39%) in our pre-COVID-19 study was disappointing. Initial invitation was by letter requiring users to type in a long URL into their digital device. There was a significant improvement in uptake when either an SMS message or email with a web-link was also sent (Supplementary Fig. 2). As might be expected, we found that the mean age of participants was lower than non-participants as getting older patients to engage in technology is sometimes a challenge.

One of the few advantages of COVID-19 has been the improved familiarity of the public with healthcare digital platforms [18]. Therefore, it is likely that similar platforms would gain a higher usage particularly as the “digital first” message gets across and as the government take steps to improve digital inclusion [19]. Furthermore, engagement should improve when digital platforms become an integral part of elective care of the patient using established lines of communication e.g. NHS App [2] rather than an invitation to participate in research.

User experience (UX) is key to the success of any digital platform and is a whole industry in itself [20]. Feedback from our users (Table 5) demonstrated several valuable lessons. Firstly, the security checks for logging in and repeated lock outs were very frustrating and deterred them from using the platform. They also found the platform was difficult to use on mobile devices. Furthermore, users complained that there were too many questions with too much repetition. The use of standardised, validated ePROMs questionnaires involved 36 generic questions and 6 specific questions. There were also 10 consent understanding questions and 17 preoperative health questions making a total of 69 questions for the initial assessment. This “question fatigue” was manifest in a high dropout rate over time [5]. With respect to informed consent metrics, true understanding can only be captured by correct answers to questions. However, indirect metrics can be captured by the system without the user having to answer questions, such as the time spent engaging with the information, the clicks onto additional information, or the playing of animations. This will improve the user experience whilst still collecting valuable metrics which demonstrate that the informed consent process has taken place.

We have listened to the user feedback from this pilot project and have developed a next-generation digital platform for sharing high quality, plain English crystal marked patient information for most medical and surgical procedures ensuring good user experience, which works on all types of devices. Additionally, gathering background metrics to demonstrate that quality information sharing has taken place to support informed consent.

Unfortunately, clinician feedback on the presence of a clinical report prior to clinic consultation and its impact on the consultation was not measured. However, patient user feedback on the digital platform did include positive comments about the quality and simplicity of the information provided, and the benefit of multimedia adjuncts such as videos and animations. The information was felt to be “easy to read and navigate”, and made users feel that they were a part of the shared decision-making process. One user stated that “The process makes me feel responsible for making a good decision about my own healthcare”.

### Future work

As digital consent solutions are implemented in healthcare, we will be measuring the impact of these healthcare technologies in terms of user experience of both clinicians and patients, user satisfaction with the informed consent process, and ultimately the impact on reducing litigation for “failure to inform”.

## Supplementary Information

Below is the link to the electronic supplementary material.

Supplementary file1 (DOCX 6733 KB)

## Data Availability

Data will be available upon reasonable request from PE (editorial@eidohealthcare.com).
