# Supplementary material for: Digital Informed Consent: Modernising Information Sharing in Surgery to Empower Patients
Source: World J Surg. 2022 Dec 3;47(3):649–57. doi: 10.1007/s00268-022-06846-w (PMC9734622; doi:10.1007/s00268-022-06846-w)
Supplement: Supplementary file 1 — Supplementary file1 (DOCX 6733 KB) [file 268_2022_6846_MOESM1_ESM.docx]

**Supplementary Document**

**
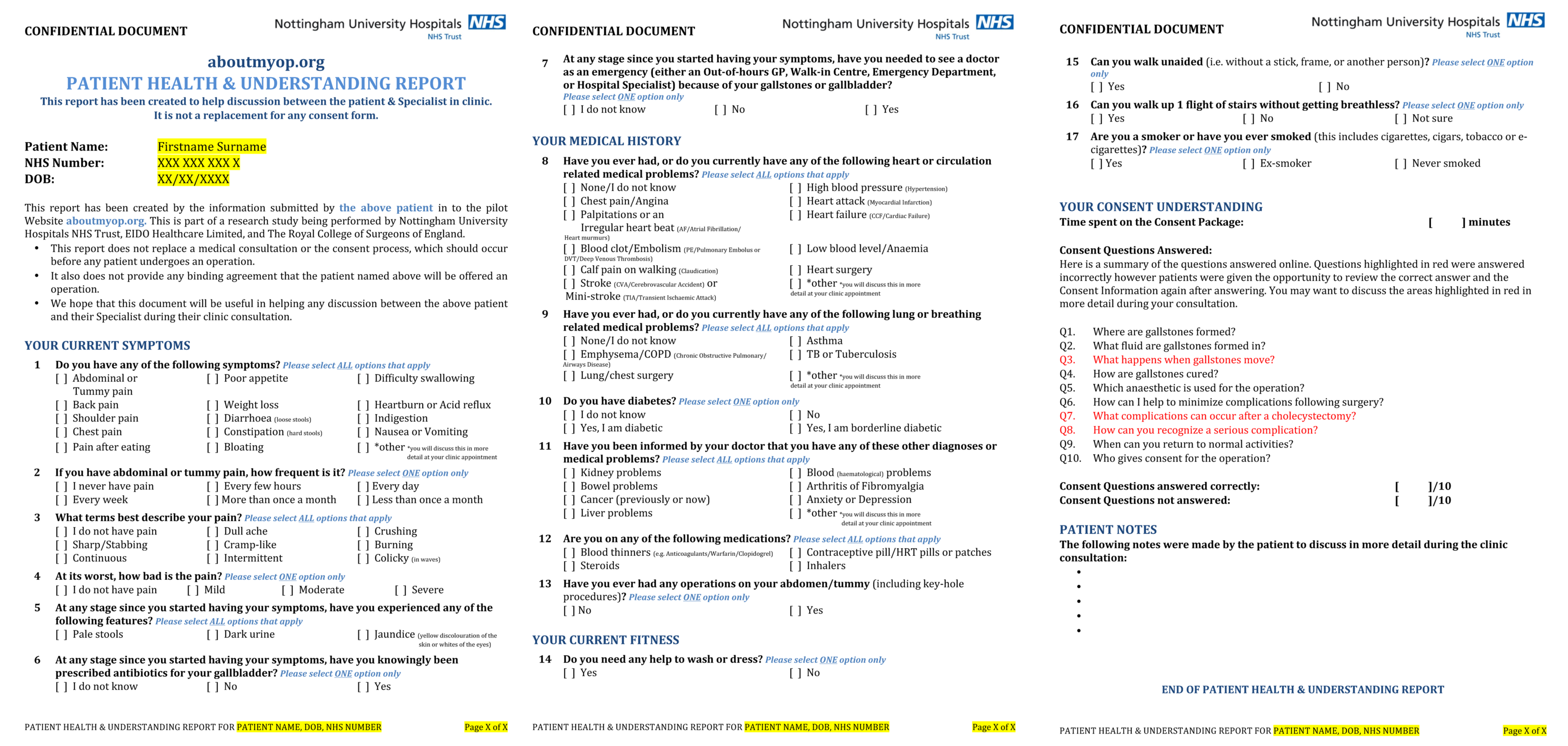
**

**Supplementary Figure 1:** Patient Health and Understanding Report (PUHR).


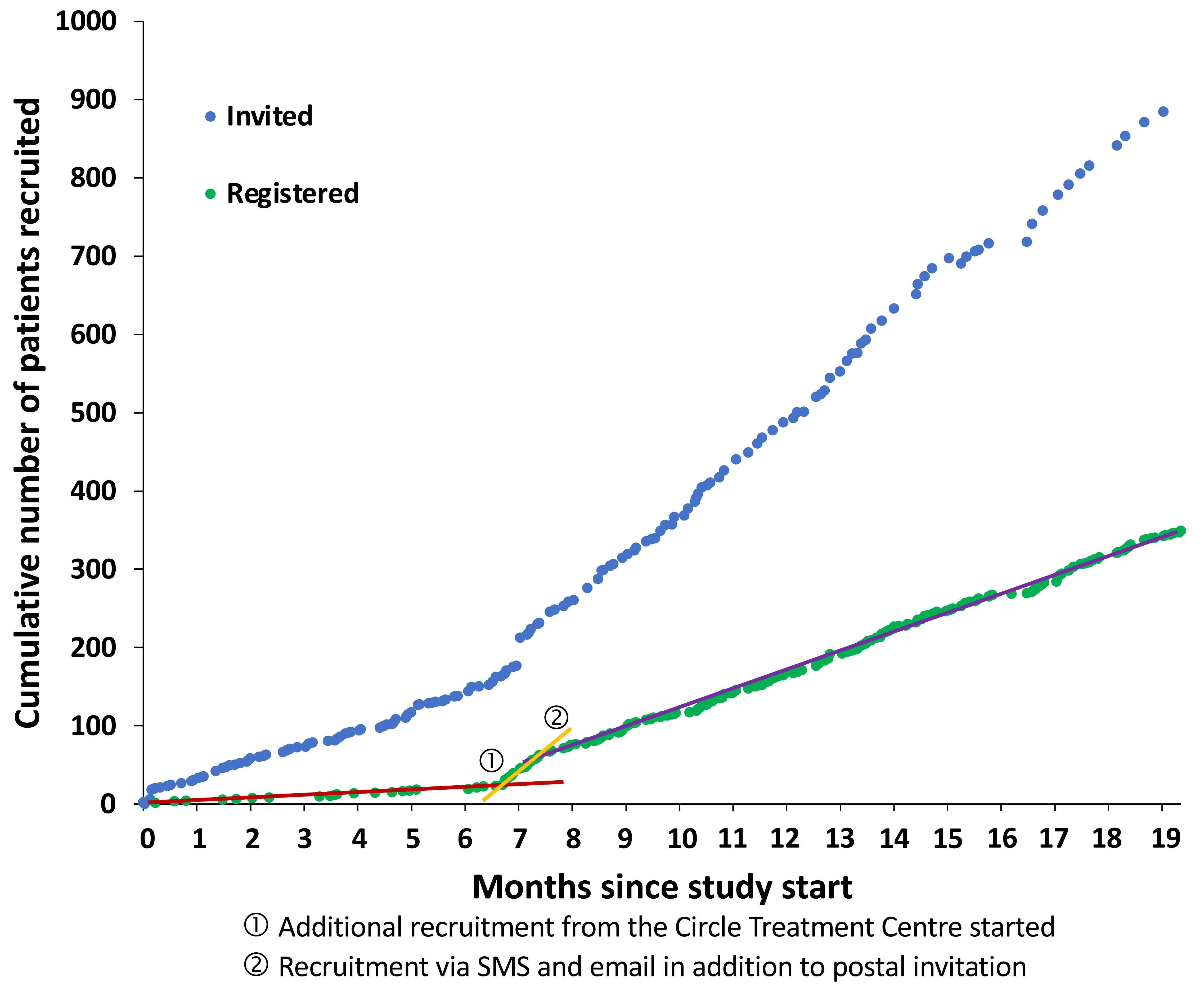


**Supplementary Figure 2:** Patient invitation vs. recruitment over study period.
